# A Scheduling Optimization Approach to Reduce Outpatient Waiting Times for Specialists

**DOI:** 10.3390/healthcare13070749

**Published:** 2025-03-27

**Authors:** Ana Moura, Micaela Pinho

**Affiliations:** 1 Department of Economics, Management and Industrial Engineering and Tourism, University of Aveiro, Campo Universitário De Santiago, 3810-193 Aveiro, Portugal; 2 Research on Economics, Management and Information Technologies, Portucalense University, R. Dr. António Bernardino de Almeida 541, 4200-072 Porto, Portugal; michaelapinho@hotmail.com

**Keywords:** outpatients waiting times, linear programming, hybrid approaches, healthcare services, prioritization-scheduling approach

## Abstract

Background/Objectives: Long waiting times for outpatient care remain a global challenge for public health systems. In Portugal, the National Health Service (NHS) ensures universal access to medical treatment, aiming to promote equity in healthcare. However, persistent delays in outpatient speciality appointments hinder this objective. Methods: This study proposes a prioritization-scheduling approach that integrates a mathematical model with a heuristic method to enhance accessibility in NHS hospitals. By optimizing the available capacity of hospitals within each geographic area, the model efficiently sequences patient appointments across different facilities, prioritizing those who have waited the longest. The approach was tested using simulated instances based on real NHS hospital data. Results: Results indicate that the model effectively integrates hospital resources within a region and efficiently allocates specialist appointments, significantly reducing waiting times. Conclusions: This research introduces a promising strategy that, when incorporated into a decision support system, can serve as a valuable tool for healthcare management.

## 1. Introduction

Outpatient waiting times pose a significant challenge for public health systems worldwide and serve as a key indicator of the quality of care provided by healthcare institutions. Efficient patient flow management is crucial in addressing these delays, as it directly or indirectly impacts patients’ health outcomes. The growing demand for healthcare services, coupled with system constraints, has led to serious consequences, drawing increasing attention in academic research. Long waiting times not only frustrate patients but also create dissatisfaction among the public and policymakers [1]. This research topic is particularly relevant in Portugal since the NHS faces significant challenges in ensuring effective healthcare access, with long waiting lists, high out-of-pocket costs, staff shortages, and increasing pressures exacerbated by the COVID-19 pandemic. These issues are partly due to a hospital-centric system that suffered from underinvestment following the global financial crisis. Between 2010 and 2013, GDP fell by 5.4%, and total health expenditure decreased by 12.4% [2].

Public health spending cuts were notable, with personnel expenses reduced by 27% (2010–2012) and capital expenses by 81% (2010–2014).

In 2021, Portugal’s health expenditure was 11.1% of GDP, aligning with the EU average, but per capita spending remained over one-third lower [3]. Portugal allocated 44% of health spending to outpatient care, the highest in the EU, contributing to low rates of preventable hospitalizations [3]. However, in 2022, 2.9% of the population faced unmet medical needs.

Given this context, implementing effective mechanisms to reduce outpatient waiting lists has become essential to maintaining the sustainability of the NHS. This study focuses on optimizing appointment scheduling to alleviate waiting times for outpatient consultations in Portuguese NHS hospitals. Despite ongoing research and policy interventions, long delays persist, particularly in the public sector, making access to specialized care a persistent challenge. The proposed approach introduces a prioritization-based scheduling optimization system designed to enhance resource allocation for medical specialists and consultation rooms. By strategically sequencing appointments across different hospitals within the same specialty, this system aims to minimize waiting lists by prioritizing patients who have endured the longest delays.

Thus, this study aims to answer the following research question: Can a prioritization-scheduling decision-making system improve resource allocation and reduce waiting times for specialist consultations in the Portuguese public sector?

Although some models have already been developed to improve access to healthcare (Section 2), no model has yet been developed for Portugal or designed to minimize access times to hospital outpatient appointments.

In Portugal, a policy instrument was introduced in 2008 to address excessive outpatient waiting times by establishing a maximum waiting-time guarantee. This measure built upon previous reforms [4], including activity-based hospital funding (implemented in 1997) and the introduction of free hospital choice for patients (enacted in 2016). The most recent policy shift, legislated in May 2016, was driven by disparities in outpatient waiting times across different specialties and NHS hospitals. This measure represented an initial step in fostering competition among NHS hospitals, as patients’ hospital choices are influenced by waiting times. As part of this initiative, a dedicated NHS website (https://www.spms.min-saude.pt/2015/12/te-m-s-tempos-de-espera-medios-na-saude/ accessed on 2 June 2022) was launched in February 2016, offering real-time updates on outpatient appointment waiting times across various specialties. This tool enables NHS users, in consultation with their general practitioners (GPs), to make informed decisions about where to seek treatment based on geographical proximity and expected response times. GPs, prioritizing patients’ best interests, can now refer them to any NHS hospital offering the required specialty, taking both location and waiting times into account. This reform marked the first step in fostering competition among NHS hospitals, as patient choice became increasingly influenced by waiting time considerations [4].

Hospitals in the Portuguese northern region had the highest levels of appointments in 2019, with waiting times above the Maximum Waiting Time Guarantee (MWTG)—39.2% (ERS, 2020). In 2019, 70% of the national average of medical appointments were carried out within the MWTG. The highest degree of compliance with the MWTG was recorded in appointments screened as a priority (75.5%), followed by high-priority appointments (73.2%) and screened appointments with normal priority (69.0%) [5].

Nevertheless, it is worth reporting that compliance with the MWTG for normal priority appointments has been decreasing since 2014 when it reached 75.3% (SNS, 2019). Although some effects of these measures have been reported [4,5], the situation has not improved regarding long outpatient waiting times for most specialties in Portugal. In fact, during the second half of 2020, neurosurgery (75%), ophthalmology (61%), orthopedics (57%), pneumology (53%), otolaryngologist (49%), and dermato-venereology (48%) recorded the longest waiting times (medical appointments with waiting times longer than the MWTG) [6].

Despite major organizational reforms in hospitals comprising new management models, the problem of long outpatient waiting still remains in Portugal. Indeed, the literature has shown that theoretical reforms or initiatives in a medical setting to enhance hospital organization for improved outcomes, without proper implementation and monitoring, often do not lead to practical improvements [7]. Although recent data show an increase in scheduled hospital activity at the level of first outpatient visits—which increased by 15.7% between 2010 and 2019 and by 2.2% between 2018 and 2019—the average waiting time for the first medical appointment increased to 83.6 days (an increase of 3.2% between 2018 and 2019). This average waiting time varies significantly between Portuguese hospitals, with records ranging from 55 days in Alentejo (south) to 90 days in the north [5].

## 2. Literature Review

Waiting time has a significant impact on patients’ overall experience and affects perceptions of quality and likeability [8]. Delays in diagnosis as a result of long waiting times may worsen health outcomes or reduce expected health gains [9]. Finally, not providing adequate and timely access to services poses a great challenge in pursuing equity—one of the main objectives of modern healthcare systems.

Lack of resources is often cited as a reason for long waiting times and queues in health services. Although the lack of material, financial, and human resources on the one hand and the growing demand for medical care on the other can influence patient waiting times, other factors such as organizational malfunctions in terms of planning and available capacity are also mentioned in the literature [10,11].

According to Allder et al. [12], a mismatch between the variation in demand and capacity is often a major cause of queuing. Furthermore, reports from the NHS Institute in Portugal show that patient admissions and completed medical appointments per consultant can vary by over 100% across hospitals in the same healthcare system [13,14]. Accordingly, the actual capacity of a given resource may depend on the skill mix and how resources are organized [15].

Operational research has been applied to the study of waiting lines and scheduling systems in outpatient departments of hospitals and in the planning of these institutions since the 1950s (see [16,17] for a review). More recently, both analytical [10], optimization, and simulation methods have been combined in solving capacity problems of healthcare [18,19,20,21] that may be useful in this context. Johannessen and Alexandersen [10] conducted a detailed examination of existing operational workflows using value-stream mapping, a technique derived from lean production principles, to pinpoint inefficiencies and bottlenecks. As a result, they successfully enhanced waiting time metrics while simultaneously extending the planning horizon. A goal programming approach was applied by Oddoye [18] to reach efficient resource planning in the medical assessment unit. The model performance was confirmed with real data, with doctors, nurses, and beds being the main resources to be allocated. Later, Oddoye et al. [22] used a goal programming and a discrete event simulation to balance the resources of a medical assessment unit and addressed the values of five performance criteria combined with the lengths of queues, the number of beds, and the waiting times.

In a private clinic context, Heshmat et al. [23] used a design thinking approach to develop a design application solution to deal with online booking and consultation. The authors proposed design features and functions to improve the quality of communication between patients and clinic staff, decrease waiting times, and develop schedules for patients and staff in order to move from a physician-oriented strategy towards a patient-oriented clinic service.

To deal with real-life problems of outpatient clinics, Lin et al. [24] proposed a novel two-stage simulation-based heuristic algorithm to assess various tactical and operational decisions for optimizing the multiple objectives—minimizing resource overtime, patient waiting time, and waiting area congestion. Using data from an ophthalmology clinic in a public hospital, the results show that the approach significantly mitigates the undesirable outcomes by integrating the strategies and increasing the resource flexibility at the bottleneck procedures without adding resources [24].

To tackle the challenges encountered by outpatient clinics, Lin et al. [24] developed a heuristic algorithm based on a two-stage simulation. This method aims to optimize key factors, such as reducing resource overtime, shortening patient wait times, and easing congestion in waiting areas. Using data from an ophthalmology clinic in a public hospital, the results show that this approach effectively minimizes adverse effects by improving resource adaptability at bottleneck procedures—without requiring additional resources [24].

By recognizing that one of the customer requirements is to reduce the waiting time to see a doctor that is in high public demand, Lee et al. [25] developed an appointment scheduling optimization model with LINGO software using the exact algorithm to be applied to the appointment scheduling problem for the outpatient clinic. To deal with the problem of appointment scheduling in outpatient chemotherapy departments, Heshmat et al. [26] developed a model based on clustering and mathematical programming that assigns every nurse to a cluster of patients and chairs on the optimum time slot to achieve the minimum completion time of the assigned nurses. The model proposed by the authors reduced the number of variables and constraints and increased the ability to give optimal solutions for real problems in reasonable computation times. Moreover, machine learning algorithms and scheduling rules were used by Srinivas and Ravindran [27] to propose a prescriptive analytics framework to improve the performance of an appointment system with respect to patient satisfaction (measured using average patient waiting time and number of patients unable to get an appointment for the day under consideration) and resource utilization (measured using average resource idle time, overflow time, and overtime).

Also in an outpatient context, Ref. [21] showed that, based on two-fold evidence, a doctor’s time is the scarcest health resource and the demand for appointments is surrounded by uncertainty. The authors recognized the difficulty of providing an exact match between the planned doctor availability and the patient’s appointment requests. Thus, the authors proposed a cardinality-constrained robust optimization model to provide a tactical capacity plan for the appointments with first-visit and revisit patients dependent on a decision maker’s risk attitude toward demand uncertainty.

Heshmat and Eltawil [28] introduced a two-phase approach to enhance scheduling in outpatient chemotherapy clinics. The overall objective is to identify the most effective scheduling practices to reduce patient wait times. In the first phase, they developed a mixed-integer linear programming model that determines the treatment start dates for new patients and optimizes all the resources needed. The second phase employs a discrete event simulation model to create patient appointment schedules aimed at minimizing treatment delays and daily completion times while adhering to several constraints.

Ordu et al. [29] developed a hybrid model that integrates forecasting, simulation, and optimization in healthcare. They connected all services and specialties within a hospital, including emergency, outpatient, and inpatient care, to predict demand for each area. Their approach utilizes discrete event simulation to address uncertainties in patient pathways through the hospital. In addition, they created a linear optimization model to determine the necessary bed capacity and staffing for a mid-sized hospital.

Nevertheless, despite this growing interest in looking for solutions to the problem of long waiting times for outpatient consultations, there are no studies that either improve the accessibility of outpatients in NHS hospitals resorting to the entire capacity of a given region nor applied to reduce outpatients waiting times in Portugal. Therefore, the present study proposes a single objective prioritization-scheduling approach that combines a mathematical and a heuristic model to increase the access of outpatients to NHS hospitals in Portugal. The approach uses the available capacity of the NHS hospitals in each geographic area and sequences patients for appointments in different hospitals by giving priority to patients who have waited longer in order to minimize waiting times. The proposed approach was tested with generated instances based on real data from the NHS hospitals. A detailed description of the model can be found in Section 3.1.

## 3. The Prioritization-Scheduling Optimization Approach

The prioritization-scheduling optimization approach aims to prioritize patients and their scheduling to improve access to services in a medical appointment planning context. All included patients are on a waiting list for a certain clinical specialty, for a given appointment, and each patient has a utility score, which indicates the days they have spent waiting for the first medical appointment. This score is randomly generated according to the average waiting time for first outpatient consultation values, made available by the NHS since February 2016 (online data on waiting times for emergency care, as well as average waiting times for outpatient consultations, detailed by specialty) [available at: https://www.spms.min-saude.pt/2015/12/te-m-s-tempos-de-espera-medios-na-saude/ accessed on 2 June 2022]. The prioritization-scheduling optimization approach is a hybrid approach where a mathematical model is combined with a heuristic that allows a decision maker to choose one or more hospitals to allocate the patients of a given clinical specialty on the waiting list to medical appointments. The mathematical model was developed based on the idea presented by Oliveira et al. [30] to assess the impact of patient prioritization when scheduled for non-urgent surgeries. The author presents a model with a multi-objective function. The process has three objectives: first, the selection of patients with higher utility scores to be scheduled sooner; second, complying with the doctors’ preferences; and third, avoiding overtime work. Based on the global idea of this model, we developed a mono-objective linear programming model applied to reduce waiting lists in an outpatient context. The main idea is to not only select patients with higher priority, but also to schedule them sooner. In this case, a patient with higher priority is a patient who has been on the waiting list for a longer period.

### 3.1. Mathematical Formulation

One way to reduce waiting lists for outpatient appointments in Portuguese NHS hospitals is to carry out efficient planning of medical appointments. To do so, the main idea is that each patient on the waiting list is assigned to hospitals of the same geographical area and according to their capacity, for each clinical specialty.

Let us assume that a planning horizon contains a set of days *D*, each day d∈D has a flexible capacity for medical appointments (number of appointments per day in the morning and in the afternoon period). There are also a set of doctors *M* of a given clinical specialty and a set of hospitals *H*. Each doctor m∈M works in a hospital h∈H, and this information is given by a Boolean matrix $lt_{mh}$. During a working day, let us assume that a doctor of a given specialty can be on duty in the emergency room, in the infirmaries, on outpatient consultations, or even in a mix of these three situations. Therefore, on each day of a planning period, the doctor can give consultations in the morning and/or in the afternoon or not make any consultations at all. So, each doctor has a number of possible appointments, per day and period, in the planning horizon, $nc_{m}$, according to their preestablished schedule. For the planning horizon, the days and periods where each doctor can give consultations is given by a Boolean matrix $hm_{md}$.

In each hospital facility, a set of doctor’s offices is also available. Each office is equipped with all the necessary materials to perform the appointment (according to the medical specialties). For a given specialty, the number of doctor’s offices available per day and period and in each hospital is given by $g_{h}$. At the beginning of the planning horizon, there is a set of patients *P* on the waiting lists. Each patient p∈P of a particular specialty has a waiting time, $wt_{p}$, which corresponds to the number of days they are waiting for a doctor’s appointment. This number of days on hold is what will dictate the priority of the appointment (the utility score previously mentioned, in Section 3). That is, the longer the waiting time, the higher the priority for scheduling the patient’s medical appointment.

The following data is a summary of what was described above.
**Model Components****Description**D={1,...,d}set of periods on planning horizons (planning horizon = number of days × two periods);H={1,...,h}set of hospitals;P={1,...,p}set of patients waiting for a consultation of a clinical specialty;M={1,...,m}set of doctors of a clinical specialty;$nc_{m}$number of doctors’ *m* appointments per period (integer value);$g_{h}$number of offices available per period and per hospital *h* (integer value);$wt_{p}$patients *p* priority (integer value);$lt_{mh}$equal 1 if the doctor *m* works in the hospital *h*, and 0 if not (Boolean auxiliary variables);$hm_{md}$equal 1 if the doctor *m* works during the period *d*, and 0 if not (Boolean auxiliary variables);

In addition to these data and auxiliary variables, the following three sets of binary decision variables are also considered in the mathematical formulation:

$$\begin{array}{c} \;\;\;\;\;\;\;\;\;\;\;x_{pmdh}=\left\{\begin{array}{c} 1,\;\mathrm{if}\mathrm{patient}p\mathrm{is}\mathrm{scheduled}\mathrm{with}\mathrm{doctor}m\mathrm{during}\mathrm{the}\mathrm{period}d\mathrm{in}\mathrm{hospital}h\\ 0,\;\mathrm{otherwise} \end{array}\right. \end{array}\;y_{mh}=\left\{\begin{array}{c} 1,\mathrm{if}\mathrm{doctor}m\mathrm{is}\mathrm{assigned}\mathrm{to}\mathrm{work}\mathrm{in}\mathrm{hospital}h\\ 0,\mathrm{otherwise} \end{array}\right.\;\;\;\;\;\;\;\;\;z_{md}\left\{\begin{array}{c} 1,\mathrm{if}\mathrm{doctor}m\mathrm{is}\mathrm{scheduled}\mathrm{during}\mathrm{the}\mathrm{period}d\mathrm{of}\mathrm{the}\mathrm{planning}\mathrm{horizon}\\ 0,\mathrm{otherwise} \end{array}\right.$$

The integer linear programming model is formulated as follows:

$$maximize\;Z=\sum_{p\in P}\sum_{m\in M}\sum_{d\in D}\sum_{h\in H}wt_{p}x_{pmdh}$$

Subject to:

(1) $$\sum_{m\in M}\sum_{d\in D}\sum_{h\in H}x_{pmdh}\leq 1;\;\forall p\in P$$

(2) $$lt_{mh}\geq y_{mh};\;\forall m\in M;\forall h\in H$$

(3) $$hm_{md}\geq z_{md};\;\forall m\in M;\forall d\in D$$

(4) $$\sum_{h\in H}\sum_{p\in P}x_{pmdh}\leq nc_{m};\;\forall m\in M;\forall d\in D$$

(5) $$\sum_{m\in M}y_{mh}\leq g_{h};\;\forall h\in H$$

(6) $$x_{pmdh}\leq y_{mh};\;\forall p\in P;\forall m\in M;\forall d\in D;\forall h\in H$$

(7) $$x_{pmdh}\leq z_{md};\;\forall p\in P;\forall m\in M;\forall d\in D;\forall h\in H$$

(8) $$x_{pmdh},y_{mh},z_{md}\in \left\{0,1\right\}$$

For each clinical specialty, the objective function prioritizes the patient’s appointment with the longest waiting times. Constraint (1) ensures that each patient is only assigned to a doctor and to a consultation in one hospital. Constraint (2) ensures that each doctor is only assigned to consultations in the hospital where he has a working schedule. Constraint (3) enforces that each doctor is only assigned to consultations in its working days and periods of the planning horizon. Constraint (4) ensures the doctors’ number of appointments for each working day and period of the planning horizon is not exceeded. Constraint (5) guarantees that the hospital’s number of available offices for consultations is not exceeded. Constraint (6) and (7) represent the linking constraints between the decision variables $x_{pmdh}$,$y_{mh}$ and $z_{md}$. Finally, Constraint (8) represents the variables’ domain.

This integer linear programming model links the prioritization system that considers patients’ waiting times to the scheduling optimization system.

### 3.2. A Hybrid Approach

The basic idea of the prioritization-scheduling optimization approach is to link the need to set priorities among patients in order to minimize their waiting times within the installed capacity of each hospital in a given geographical region. To be able to do that, this approach should be connected to the databases of all national hospitals. This way, whenever an appointment is made, the respective hospital capacity and the NHS waiting lists will be automatically updated.

The hybrid approach is designed to be used in a hospital for the management of their patients’ appointments. However, as it turns out, in reality, hospitals do not always have the capacity to attend (in a timely manner) to all patients. Therefore, the developed approach (Algorithm 1) always gives priority to scheduling hospital appointments according to their capacity. If a hospital does not have sufficient capacity for patient appointments during a specified planning period, the decision maker will attempt to schedule the patients at one or more other NHS hospitals within the same geographical area and clinical specialty. To do this, the decision maker must have access to information regarding the doctors, specialties, and capacities of each hospital. This means that they need to be able to access the databases of all relevant hospitals. The algorithm (Algorithm 1) starts with the decision maker selecting the hospital, medical specialty, and the planning horizon to be scheduled. After that, the algorithm checks if there is available capacity in the selected hospital for the chosen planning horizon to schedule the patients, either in terms of doctors with free time for appointments and doctor’s offices. Then, the integer linear programming model is utilized.

The model returns a solution that is a schedule of the doctor/patient and the date and time of the consultations. Then, the algorithm checks if there are still patients on the waiting list (for the same specialty) that could not be scheduled due to the lack of capacity. If that is the case, the decision maker chooses another hospital in the same geographical area that provides the same specialty. For the chosen hospital(s), the capacity is checked in terms of doctors with free time for appointments and doctor’s offices for the planning horizon. If there is enough capacity to schedule more consultations, the model is called, and a solution is found, giving all the scheduled appointments and related hospitals and doctors. However, if a solution is not found (if there is no valid solution), the decision maker will be questioned if they want to select another hospital.
**Algorithm 1** Hybrid approach1:**Start:** Medical Specialty *m*, planning horizon *p*, hospital(s) *h*2:**Output:** Patients Scheduling *S*3:cap←m;p;h4:Capacity Availability cap5:**while** cap≥0 **do**6:    Mathematical model S7:    **if** waitinglist==0 Or validsolution **then**8:        *S*9:    **else**10:        Chose another hospital11:    **end if**12:**end while**13:**return** *S*

The algorithm ends when any of the following situations append: (i) whenever the decision maker decides not to choose another hospital, (ii) if there are no more patients on the waiting list, and/or (iii) if there are no longer available capacity for the planning horizon in all the hospitals and for the medical specialty.

## 4. Numerical Experiences and Test Results

In this paper, the authors intend to assess how optimization tools might improve the accessibility for outpatients to specialists in Portuguese NHS hospitals by reducing long waiting times across a range of specialties. Therefore, we simulate the problem using real data of average waiting times for outpatient consultations (by specialty) in four NHS hospitals in the north of the country this data (available at https://www.spms.min-saude.pt/2015/12/te-m-s-tempos-de-espera-medios-na-saude/ accessed on 2 June 2022) is made available by the NHS and updated in real-time). We used the last available data ranging from April to June 2020 (a very complicated year due to COVID-19) for six specialties that have, according to the Health Regulator (ERS), recorded longer waiting times, namely neurosurgery, orthopedics, ophthalmology, pneumology, otolaryngologist, and dermato-venereology [6].

Based on these recorded data, some test instances were generated and tested using the mathematical model presented in Section 3.1.

The problem instances include data for a medical specialty of a given NHS hospital and the following information is required:

- The number of patients waiting for a medical appointment (patients waiting list size);
- Average waiting time (in days) for each specialty (or per waiting list);
- Number of specialist doctors;
- Installed capacity of hospitals—number of offices available for outpatient appointments.

Despite having real data, concerning the first two requirements, namely the number of patients in waiting and the average waiting time (in days), there is a lack of information concerning:

- Patients’ individual waiting time;
- Number of specialist doctors in each hospital allocated to the consultations during each planning period;
- Average time spent per medical appointment;
- The number of offices for outpatient appointments.

To overcome the problem of this missing information, these data were generated randomly, within reasonable values, and as close as possible to reality. The duration for each medical appointment was set as 20 min. The planning period (for medical consultations) is variable, but always considered five working days per week. As mentioned before, this planning period is always given by the number of days, times two consultation periods (morning and afternoon). A doctor could be available for appointments for one or two periods per day (morning or afternoon) and during five or fewer working days per week. In the absence of information on the subject, we assume that the number of offices for outpatient appointments per hospital and specialty is always equal to the available doctors each day.

According to the historical records, depending on the hospital and specialty, the waiting list size could vary from 91 to 4984 patients and the waiting days can vary from 79 to 432. In the real data, we have no information (for reasons of privacy and data confidentiality) on the number of days that each patient is waiting for the first appointment. So, this value is randomly generated according to the average number of waiting days for a particular specialty.

Thus, for each patient on a specialty waiting list, a number between one and the related average number of waiting days is randomly generated. This will be the actual number of days that a patient is waiting for the first medical appointment.

The machine used to run all the numerical tests was an Intel (R) Core (TM) i7-4750HQ CPU @ 2.00 GHz computer with 8.00 GB of RAM and the Windows 10 Home operating system. The mathematical model was implemented using the solver CPLEX Studio IDE 12.6.3, and a hybrid approach was implemented in Phyton.

To test the mathematical model, a sensitivity analysis was performed (Section 4.1), assuming four and six hospitals and one specialty. According to the hybrid approach, presented in Section 3.2, two types of problem instances were considered: (i) a group of instances using data from one hospital with six specialties and (ii) a group of instances assuming the same six specialties but using data from four NHS hospitals in the north of the country.

### 4.1. Mathematical Model Behavior

The idea is to test the model to verify its effectiveness and robustness. To this end, 24 test problems were created with sufficiently different data, so that a sensitivity analysis of the model could be performed. Two classes of problems were created, I3 and I4. Each class of problems is based on real data from two clinical specialties: Class I3, for the otolaryngologist specialty, and Class I4, for the ophthalmology specialty. In the first class of four hospitals, a five-day planning period and an average waiting time of 196 days are considered. In the second class, there is a bigger set of problem instances, with six hospitals, a ten-day planning period, and an average waiting time of 284 days considered.

Each class has three different types of instances (I31, I32, I33 and I41, I42, I43). For each class, the differences between the three types of instances are related to the number of specialist doctors available in the hospitals (varies from 18 to 22, for class I3; and from 10 to 16, for class I4), the number of patients on the waiting lists (varies from 700 to 1000, for class I3; and from 1000 to 1400 for class I4). The instances I31 and I32 and I41 and I42, have an average number of offices (per hospital) of 4.5, being evenly distributed, while I33 and I43 have a 2.5 average and a non-even distribution.

In order to study which are the most critical data of the model, several problem tests were created for each type of instance, by varying the following data:

- Number of available offices for outpatient appointments (I31.1, I32.1, I33.1 and I41.1, I42.1, I43.1). For the first two instance types (I31, I32 and I41, I42), the average number of offices were reduced to a mean value of 3.5, with a more heterogeneous distribution of the number of offices per hospital; for the last ones (I33.1 and I43.1), the average of 2.5 was kept, but the distribution heterogeneity was increased.
- Number of days and related periods of doctors’ availability (I31.2, I32.2, I33.2 and I41.2, I42.2, I42.2). For each class, this value can vary between 1 and 10, and in each instance type, the number of working periods for each doctor is reduced by 12%.
- Number of appointments per day and period for each doctor according to its working scheduling (I31.3, I32.3, I33.3 and I41.3, I41.3, I41.3). For each class, this value can vary between two and eight appointments, and in each instance type, the number of appointments for each doctor is reduced by 20%.

Table A1 in the Appendix A summarizes the main results, and it provides information for a results discussion regarding the data variation. Nevertheless, it can be seen that the model always reaches an optimal solution in a very low computational time. As expected, whenever the number of available offices for outpatient appointments decreases by an average of 30%, the computational time (CPU) increases significantly. Moreover, for the same number of doctors, whenever the number of working days decreases by 12%, the CPU time is slightly lower. However, this CPU difference is not significant whenever the doctors’ number of appointments per day is reduced by 20%, ensuring that the number of specialist doctors and the number of their working days remains constant.

A robustness test was also performed to evaluate the growth of the CPU time increasing the size of the problem instances. Assuming one specialty, a planning horizon of 22 days, 24 doctors with total availability in this planning period, 4 NHS hospitals, and a waiting list covering up to 4000 patients, the medium CPU time needed to reach an optimal solution was 130 s. However, for problems with the waiting list covering between 4000 and up to 8000 patients the medium CPU time increases to 670 s.

We can conclude that the model is quite efficient and the computational times are not very high, even for relatively large problems. However, for long waiting lists, when increasing the number of hospitals and doctors in each of the hospitals, this way of resolution, by itself, is no longer efficient due to the time needed to reach a solution. This is why the heuristic approach was proposed.

### 4.2. Hybrid Approach Performance

To assess the behavior of the proposed approach, two groups of instances were created. All instances assume a planning horizon of 22 days. The first group of instances (I1: from I11 to I16) included 4 available NHS hospitals (H1 to H4), all of them with available capacity to schedule patients, 6 specialties, and an average waiting day, per specialty, of 202, 306, 242, 264, 313, and 319. The second group of instances (I2: from I21 to I26) has the same 6 specialties, and an average waiting day, per specialty, of 196, 212, 285, 245, 267, and 275.

The differences between the two groups are related to the number of available specialist doctors in each hospital for each specialty, with an average of 15 for I1 and 40 for I2 (16, 25, 19, 11, 15, 4 for I1 and 24, 49, 46, 23, 55, 41 for I2); and the number of patients in the waiting lists for each specialty was an average of 2050 for I1 and 6215 for I2 (639, 3017, 4202, 803, 2128, 1780 for I1 and 3534, 10,886, 3738, 3738, 8304, 7090 for I2).

All these data are real and made available online by the NHS. For the remaining necessary data not available in the NHS information, it was assumed that:

- The number of available medical offices for the planning period is the same as the available doctors;
- The number of doctors on duty, per day, is at least half the number of available doctors;
- Doctors only make half their working day available for consultations (corresponding to eight medical appointments);
- For the number of waiting days of each patient on the waiting list, a random number between 1 and the real number of waiting days was generated.

The approach gives priority to scheduling the appointments in H1 (the host hospital of the application). The results are presented in Table 1. Looking at the results, the model always reaches the optimal solution in a low computational time, even with the larger instances (Group I2).

For I11, I12, I14, I15, I21, and I24, all patients on the waiting list were scheduled at the hospital H1, and the necessary capacity was sufficient to schedule appointments. This scheduling always ensures that patients who have been waiting the longest are the first to have an appointment. Thus, in the worst case, a patient will have to wait for their appointment for a maximum of 22 more days (to the next planning horizon).

For the other instances, it was not possible to schedule all the patients in H1, because the hospital capacity was not enough, so it was necessary to send them to another hospital (or hospitals). According to hospital capacity and to schedule the remaining patients, three more hospitals were needed for I13 and I26, two more hospitals were needed for I16, I22, and I25, and one more hospital was needed for I23 (Table 1). This means that, for example, in problem I16, in order to schedule appointments for the specialty in question for all patients on the waiting list, it was necessary to use the services of two other hospitals: H2 and H3. With H2 also having also exhausted all its capacity, it was necessary to turn to H3. Only with these three hospitals is it possible to schedule all appointments on hold.

For each patient, the approach output provides information concerning: the hospital where the appointment will be made, the day and time, the doctor, and the medical office.

The previous tests were developed as a simulation of a real scenario, using real data mixed with generated one. It should be noted that in a real scenario after the current period was scheduled, there will be more new outpatients who have entered the system for appointments. Those patients must be considered for scheduling in the next planning horizon. This system is dynamic in line with the NHS itself.

## 5. Conclusions, Limitations, and Future Work

The prioritization-scheduling approach presented here is both timely and useful. This tool can be applied broadly to guide fair and ethical resource allocation, but it is especially relevant to improve accessibility for outpatients in Portuguese NHS hospitals. Despite numerous health policies implemented during recent years to grant equity in access to outpatient consultations, the results remain far below expectations. In this sense, the presented prioritization approach shows us an efficient and optimized way to reduce waiting times for outpatient appointments. The model proves to be effective in terms of appointment allocation per doctor and the decrease in patient waiting. Moreover, the model is innovative because it integrates hospital resources in a region. Currently, our study indicates that a substantial reduction in WT can be achieved through an integrated use of hospital resources in regions of the country. Thus, our findings provide a clear answer to our research question, demonstrating that appointment scheduling optimization can significantly reduce outpatient waiting times in Portuguese NHS hospitals.

We can also guarantee, based on this last statement, that there would be patients who would wait much less time if they could be seen at another hospital in their area of residence. However, the prioritization model proves to be effective from the theoretical point of view. Our analysis was carried out by using not only real historical data but also assuming some missing data.

Related to limitations and future research, it would be interesting to use and test the model with real data in order to validate the model. Decision making in healthcare is inherently complex, involving both trade-offs within the equity itself and between equity and efficiency. Striking a balance between these competing objectives requires nuanced policy decisions, such as integrating need-based criteria into resource allocation models or applying ethical frameworks that account for both efficiency and fairness. Keeping this in mind, a limitation of this approach is that patients are assigned appointments solely based on their waiting times. This can lead to situations where patients with more serious health problems are pushed aside in favor of those with longer waiting times but less severe conditions. Similarly, prioritizing treatment for a patient who has waited longer can disadvantage another patient who has waited less but is more likely to benefit from the intervention. Although the main idea of the model presented is to first allocate the patients with the longest waiting time, it can also be used to prioritize patients according to, for example, the severity of their health condition. With this in mind, the objective function Section 3.1 can be written as (Equation (9)):

(9) $$maximize\;Z=\sum_{p\in P}\sum_{m\in M}\sum_{d\in D}\sum_{h\in H}Ps_{p}x_{pmdh}$$

In this model, $Ps_{p}$ represents a priority score assigned to patients based on the severity of their health conditions. The variable $x_{pmdh}$ indicates whether a patient is scheduled with a specific doctor during a designated time period and at a particular hospital. This modification ensures that the model prioritizes patients with more severe health conditions.

Thus, the presented model could be applied to other healthcare rationing situations, such as medical exams or even the allocation of patients to intensive care units. We are confident that the model will effectively reduce waiting times. If it is integrated into a decision support system for hospital resource management, it could significantly enhance the healthcare system. Due to several confidential reasons, the model was validated using partial real data instances, as mentioned in Section 4. In future work, we plan not only to integrate the model with real databases but also to validate it using real-time data. Nevertheless, we acknowledge the practical challenges of implementing the developed model in real hospital settings, particularly regarding administrative constraints and data-sharing agreements between hospitals. In this context, it is the responsibility of policymakers to take the necessary steps to ensure the seamless exchange of patients’ clinical information across all SNS healthcare units.

## Figures and Tables

**Table 1:** Hybrid approach test results.

Instances	CPU Time (s)	GAP (%)	Patients not Attended in H1 (%)	Support Hospitals
I11	1.83	0	0	-
I12	55.67	0	0	-
I13	120.47	0	22	H2, H3, H4
I14	1.05	0	0	-
I15	16.34	0	0	-
I16	2.72	0	40	H2, H3
I21	83.67	0	0	-
I22	1586.83	0	54	H3, H4
I23	96.37	0	10	H2
I24	100.42	0	0	-
I25	895.32	0	35	H2, H4
I26	734.10	0	40	H2, H3, H4

## Data Availability

The data presented in this study are available on request from the corresponding author.

## Appendix A

**Table A1:** Mathematical model results.

Instances	CPU Time (s)	GAP (%)	No. of Variables	No. of Constraints	No. of Patients Attended in the Planning Period	% of Unattended Patients
I31	2.25	0	840,198	841,298	840	16
I31.1	49.41	0	840,198	841,298	560	44
I31.2	1.5	0	840,198	841,298	728	27
I31.3	2.36	0	840,198	841,298	670	33
I32	1.59	0	352,189	705,112	800	0
I32.1	48.41	0	352,189	705,112	640	16
I32.2	1.01	0	352,189	705,112	768	3
I32.3	1.63	0	352,189	705,112	710	9
I33	1.13	0	252,162	504,956	700	0
I33.1	23.97	0	252,162	504,956	600	10
I33.2	0.91	0	252,162	504,956	624	7.6
I33.3	1.08	0	252,162	504,956	580	12
I41	4.85	0	600,166	1,201,266	480	52
I41.1	60.91	0	600,166	1,201,266	406	59
I41.2	3.15	0	600,166	1,201,266	438	56
I41.3	4.74	0	600,166	1,201,266	426	57
I42	5.19	0	720,160	1,441,466	800	33
I42.1	52.72	0	720,160	1,441,466	625	48
I42.2	4.79	0	720,160	1,441,466	786	35
I42.3	5.01	0	720,160	1,441,466	723	40
I43	7.05	0	1,344,256	2,689,822	1280	9
I43.1	34.48	0	1,344,256	2,689,822	1200	14
I43.2	6.35	0	1,344,256	2,689,822	1108	21
I43.3	6.87	0	1,344,256	2,689,822	909	35
